# Study on the Protective Mechanism of Dihydromyricetin Against Aflatoxin B_1_-Induced Injury in Madin–Darby Canine Kidney Cells

**DOI:** 10.3390/cimb47110947

**Published:** 2025-11-13

**Authors:** He Zhai, Liuwei Xie, Baoan Li, Mingqiang Song, Xiao Li, Shu Xu, Yao Wang, Chao Xu

**Affiliations:** 1 School of Police Dog Technology, Criminal Investigation Police University of China, Shenyang 110035, China; 2018990121@cipuc.edu.cn (H.Z.); xielw195@163.com (L.X.); 17852028632@163.com (B.L.); song15263936064@163.com (M.S.);; 2 College of Animal Science and Technology, Jilin Agricultural University, Changchun 130118, China

**Keywords:** dihydromyricetin, aflatoxin B_1_, MDCK cells, cell apoptosis, protective effect

## Abstract

Aflatoxin B_1_ (AFB_1_) is a common contaminant in canine diets that can cause significant damage to metabolic organs with prolonged exposure. Dihydromyricetin (DMY), a flavonoid compound abundant in *Ampelopsis grossedentata*, is widely used in functional foods due to its diverse biological activities. This study aimed to investigate the mechanism by which DMY alleviates AFB1-induced damage in MDCK cells. Four experimental groups were established: a control group with culture medium only (CON group), a group treated with 5 μg/mL AFB1 (AFB1 group), and two treatment groups treated with 5 μg/mL AFB1 combined with either 25 mmol/L or 50 mmol/L DMY—concentrations with more robust and stable protective effects than 100 mmol/L DMY, as confirmed by experimental screening. The results showed that AFB_1_ significantly reduced MDCK cell viability at concentrations of 5–30 μg/mL (*p* < 0.01), while DMY at 25–100 mmol/L markedly improved cell viability (*p* < 0.01). AFB_1_ treatment led to a significant increase in reactive oxygen species (ROS), malondialdehyde (MDA), tumor necrosis factor alpha (TNF-α), interleukin-6 (IL-6), and interleukin-1β (IL-1β) levels, along with a reduction in superoxide dismutase (SOD) and catalase (CAT) activities (*p* < 0.01). 25 mmol/L and 50 mmol/L DMY treatment reversed these effects, decreasing ROS, MDA, TNF-α, IL-6, and IL-1β levels while increasing SOD and CAT activities (*p* < 0.01). Furthermore, 25 mmol/L and 50 mmol/L DMY improved mitochondrial membrane potential (*p* < 0.01), counteracting AFB_1_’s inhibitory effects on autophagy-related proteins by promoting p-AMPK and Beclin-1 expression while inhibiting p-mTOR, p53, and p62 expression (*p* < 0.05). In conclusion, DMY mitigates AFB_1_-induced damage in MDCK cells by enhancing anti-inflammatory and antioxidant defenses and promoting autophagy, providing a theoretical foundation for future treatment strategies for canine kidney damage.

## 1. Introduction

Dogs are recognized as important companion animals, contributing to human society through their roles in both labor and emotional support [1]. With the rapid expansion of the global pet industry in recent years, public awareness of companion animal health has increased significantly. Consequently, greater attention has been paid to the quality and safety of canine diets. Commercial dog foods, primarily composed of compound feeds, have gained wide acceptance in the market. Cereals rich in carbohydrates, such as corn, rice, sorghum, and wheat, are commonly included in these formulations due to their cost-effectiveness and adequate nutritional value. However, these cereals are susceptible to contamination by toxigenic fungi during cultivation, harvest, storage, and transportation, which can result in the production of mycotoxins, particularly aflatoxins [2,3,4,5].

Aflatoxins are secondary metabolites primarily produced by *Aspergillus flavus* and *Aspergillus parasiticus* [6]. Among the known aflatoxin derivatives, AFB_1_ is the most toxic, classified by the International Agency for Research on Cancer (IARC) as one of the strongest carcinogens, with properties of toxicity, teratogenicity, carcinogenicity, and mutagenicity [7]. Prolonged consumption of AFB_1_-contaminated food can significantly impair the normal function of multiple organs and tissues, including the kidneys [8,9], and lead to oxidative stress, inflammation, and cell apoptosis [10]. A previous study has shown that excessive exposure to aflatoxins can cause various macroscopic and microscopic renal lesions, such as multifocal hemorrhages and degeneration and necrosis of tubular epithelial cells [11]. AFB_1_ has also been shown to reduce cell viability and trigger apoptosis by inducing oxidative stress and inhibiting oxidative phosphorylation in renal cells, leading to increased ATP consumption and decreased ATP production [12]. These findings suggest that excessive AFB_1_ intake can promote renal inflammation and potentially lead to kidney failure. Given the unavoidable use of these feed ingredients in commercial canine diets, it is crucial to identify natural active compounds that can mitigate AFB_1_-induced kidney damage in dogs.

*Ampelopsis grossedentata* (Hand.-Mazz.) W.T. Wang is a dual-purpose plant with both medicinal and edible applications, traditionally recognized for its effects in clearing heat, eliminating dampness, detoxifying, and reducing swelling [13]. The leaves and stems of *A. grossedentata,* commonly referred to as vine tea, have been consumed as a health drink for centuries [14]. Dihydromyricetin (DMY), also known as ampelopsin, is the predominant flavonoid in *A. grossedentata,* with its content reaching up to 30% in the leaves [15]. DMY is an abundant resource, relatively inexpensive, and easy to prepare. Due to its antioxidant, anti-inflammatory, and neuroprotective properties, it is widely used in functional foods [16,17,18]. Research by Wu et al. demonstrated that DMY inhibited apoptosis in cisplatin-induced human renal cortical proximal tubule epithelial cells by upregulating the anti-apoptotic protein Bcl-2 [19]. Additionally, DMY significantly enhanced superoxide dismutase (SOD) activity and reduced malondialdehyde (MDA) levels, along with the mRNA expression of inflammatory factors such as interleukin-1β (IL-1β), interleukin-6 (IL-6), tumor necrosis factor-α (TNF-α), and monocyte chemoattractant protein-1 (MCP-1) in mouse kidneys [19]. Similar findings by Wang et al. indicated DMY’s protective effects against lipopolysaccharide-induced acute kidney injury in rats, showing a significant reduction in inflammatory markers, MDA levels, and blood urea nitrogen [20]. These studies highlight the efficacy of DMY in combating oxidative stress and inflammation in renal cells. However, the effects of DMY in alleviating AFB_1_-induced renal injury in dogs remain underexplored. Therefore, the present study aimed to investigate the regulatory effects of DMY on the anti-inflammatory and antioxidant responses in AFB_1_-induced canine renal tubular epithelial cells, determining the optimal dosage and safety of DMY. This research will provide a theoretical foundation for further validation of DMY as a treatment for inflammation and apoptosis in the canine kidney.

## 2. Materials and Methods

### 2.1. Experimental Materials

Madin–Darby Canine Kidney (MDCK) cells (Cat# CL-0154) used in the present study were obtained from Wuhan Punuosai Life Technology Co., Ltd. (Wuhan, China). AFB_1_ (≥98% purity, Cat# A6636) and DMY (≥98% purity, Cat# A0049) were purchased from Sigma-Aldrich (St. Louis, MO, USA) and Chengdu Must Biotechnology Co., Ltd. (Chengdu, China), respectively. DMEM high glucose medium (Cat# 11965092) and fetal bovine serum (Cat# A5256701) were obtained from Invitrogen (Carlsbad, CA, USA). Apoptosis was evaluated using an Annexin V-FITC/propidium iodide (PI) apoptosis detection kit (Cat# 559763; Becton-Dickinson, MD, USA). Mitochondrial membrane potential assay kit with JC-1 (Cat# C2006), DAPI (Cat# C1002), and Hoechst cell apoptosis staining kit (Cat# C0003) was purchased from Beyotime Institute of Biotechnology (Shanghai, China). The malondialdehyde (MDA) assay kit (Cat# A003-1-2), catalase (CAT) assay kit (Cat# A007-1-1), SOD assay kit (Cat# A001-1-2), were purchased from Nanjing Jiancheng Bioengineering Institute (Nanjing, China). The TNF-α assay kit (Cat# JL22455), IL-6 assay kit (Cat# JL22371), and IL-1β assay kit (Cat# JL22367) were obtained from Shanghai Jianglai Biotechnology Co., Ltd. (Shanghai, China). The CCK-8 assay kit (Cat# A-PJ1174-100T) was acquired from Shanghai Fusheng Industrial Co., Ltd. (Shanghai, China).

### 2.2. Experimental Design

#### 2.2.1. Screening of Effective Concentrations of AFB_1_ and DMY

Trial one consisted of two single-factor randomized sections, each with six replicates per treatment. In the first section, MDCK cells were incubated for 48 h in culture media containing 0, 0.5, 1, 5, 10, 20, or 30 μg/mL AFB_1_ to determine the optimal concentration that induces cellular damage, based on cell viability. In the second section, using the optimal AFB_1_ concentration identified previously, MDCK cells were co-treated with 0, 10, 25, 50, or 100 mmol/L DMY for 48 h. The objective was to identify the most effective DMY concentration for protecting MDCK cells by evaluating cell viability. In this study, DMY was dissolved in dimethyl sulfoxide (DMSO) with the final DMSO concentration in DMEM medium strictly controlled at ≤0.1% (*v*/*v*), and preliminary CCK-8 assays had verified that this DMSO concentration exerted no significant toxicity on MDCK cells; the pH of the culture medium was monitored during the entire incubation period and remained stable at 7.2 to 7.4.

#### 2.2.2. Mechanistic Study of DMY-Mediated Repair of AFB_1_-Induced Damage in MDCK Cells

Trial two was conducted using a single-factor randomized design with four treatment groups: a blank control group (CON) and three experimental groups treated with 5 μg/mL AFB_1_ alone (AFB_1_ group), or in combination with either 25 or 50 mmol/L DMY. Each group included six replicates. MDCK cells were seeded at a density of 2.5 × 10^5^ cells/mL in 24-well plates (2 mL/well) and cultured in DMEM at 37 °C until cell adhesion was achieved. The medium was then replaced, and incubation continued for 48 h. This experiment aimed to investigate the mechanism by which DMY mitigates AFB_1_-induced damage by comparing the reparative effects of different DMY concentrations based on physiological and biochemical indicators in MDCK cells.

### 2.3. Indicator Detection

#### 2.3.1. Cell Viability

Cell viability was assessed using the CCK-8 assay kit. In brief, after 48 h of treatment, 10 μL of CCK-8 solution was added to each well, and the plates were incubated at 37 °C for 3 h. Absorbance was subsequently measured at 450 nm using a Spectra Max Gemini XPS/EM spectrophotometer (Molecular Devices Corp., San Jose, CA, USA).

#### 2.3.2. Inflammatory and Oxidative Indicators

The adherent cells were gently washed with PBS and dissociated using 0.25% trypsin. The cells were then collected by centrifugation at 1000× *g* for 5 min, resuspended in PBS, and sonicated to obtain the cell extract. The extract was subsequently centrifuged at 1500× *g* for 10 min at 4 °C, and the resulting supernatant was collected for further analysis. The inflammatory factors IL-6, IL-1β, and TNF-α in MDCK cells were quantified using the enzyme-linked immunosorbent assay (ELISA) method, following the manufacturer’s instructions. Absorbance was measured at 450 nm using a microplate reader, and the levels of the inflammatory factors were calculated based on the standard curve.

Oxidative stress in MDCK cells was assessed by measuring the levels of SOD, CAT, and MDA. Briefly, treated cells were harvested and lysed, and the lysates were centrifuged at 12,000 rpm for 10 min to collect the supernatant. SOD, CAT, and MDA levels were then quantified using commercially available assay kits, following the manufacturer’s protocols. SOD activity was determined by the hydroxylamine method, with absorbance measured at 550 nm. CAT activity was assessed using the ammonium molybdate method, and absorbance was recorded at 405 nm. MDA concentration was quantified via the thiobarbituric acid (TBA) method, with spectrophotometric detection at 532 nm.

#### 2.3.3. Hoechst 33258 Staining and Mitochondrial Membrane Potential

After 48 h of culture, MDCK cells from each group were digested with 0.25% trypsin and centrifuged at 1000× *g* for 5 min. The supernatant was discarded, and the cells were washed twice with PBS, then collected by centrifugation at 2000× *g* for 5 min. A 4% tissue fixative solution was added to fix the cells. After fixation, the fixative was removed, and 1 mL of Hoechst 33258 staining solution (10 μg/mL in PBS) was added to each well. The cells were incubated in the dark at 37 °C for 10 min, then washed twice with PBS. Finally, the cells were observed under a fluorescence microscope using excitation at 350 nm and emission at 460 nm. For each group, ten random fields of view were selected and counted. Cells showing nuclear condensation and fragmentation were identified as apoptotic.

The JC-1 mitochondrial membrane potential assay kit was used to assess mitochondrial membrane potential in MDCK cells [21]. After 48 h of culture, the medium was removed, and 1 mL of JC-1 working solution was added to each well. Cells were incubated at 37 °C for 20 min, then washed twice with JC-1 staining buffer. Subsequently, 1 mL of DAPI working solution (1 μg/mL) was added and incubated in the dark for 10 min. After two washes with PBS, cells were observed and imaged using a fluorescence microscope. Regions with high mitochondrial polarization showed red fluorescence, while depolarized regions showed green fluorescence. Mitochondrial membrane potential was expressed as the ratio of red to green JC-1 fluorescence.

#### 2.3.4. Flow Cytometry

To assess cell apoptosis, 100 μL of buffer was added to MDCK cells cultured for 48 h, and the cells were gently resuspended. Next, 5 μL of FITC-Annexin V and 5 μL of PI working solution were added, and the mixture was incubated in the dark at room temperature for 15 min. The cells were then washed with binding buffer, centrifuged, and the supernatant was discarded. The cell pellets were then resuspended in 400 μL of binding buffer and analyzed by flow cytometry. In a separate experiment, the same procedures were followed, except that cells were incubated with 2′,7′-dichlorodihydrofluorescein diacetate (DCFH-DA) probe for 30 min at 37 °C in the dark to assess intracellular reactive oxygen species (ROS). After incubation, cells were centrifuged, and the resulting pellets were collected and transferred to flow cytometry tubes for analysis.

#### 2.3.5. Western Blot

MDCK cells cultured for 48 h were harvested and lysed using RIPA buffer supplemented with protease and phosphatase inhibitors. The lysates were centrifuged at 3000 rpm for 5 min, and the supernatants were collected. Equal amounts of protein were mixed with loading buffer and subjected to SDS-PAGE. Following electrophoresis, proteins were transferred onto nitrocellulose membranes. The membranes were blocked with 5% non-fat dry milk and incubated overnight at 4 °C with primary antibodies (Cell Signaling Technology, Inc., Danvers, MA, USA) against p62 (1:1000, Cat# 5114), AMPK (1:1000, Cat# 2532), p-AMPK (1:1000, Cat# 2531), mTOR (1:1000, Cat# 2983), p-mTOR (1:1000, Cat# 2971), Beclin-1 (1:1000, Cat# 3738), p53 (1:1000, Cat# 9282), and GAPDH (1:2000, Cat# 2118). After washing, membranes were incubated with appropriate secondary antibodies for 1.5 h at room temperature. Signal detection was performed using ECL reagents, and protein band intensities were quantified using ImageJ software (version 1.8.0; ImageJ Software Inc., Washington, MD, USA). Western blotting was performed in triplicate.

### 2.4. Data Analysis

Data were analyzed using the MEANS procedure in SAS 9.4 software (SAS Institute Inc., Cary, NC, USA). One-way analysis of variance (ANOVA) was performed using the GLM procedure, followed by Duncan’s test for post hoc comparisons. Statistical graphs were generated using GraphPad Prism 8.0 software (GraphPad Prism Inc., San Diego, CA, USA). *p* < 0.05 was considered statistically significant.

## 3. Results

### 3.1. Screening of AFB_1_ Damaging Concentration and DMY Protective Concentration

**Figure 1:**
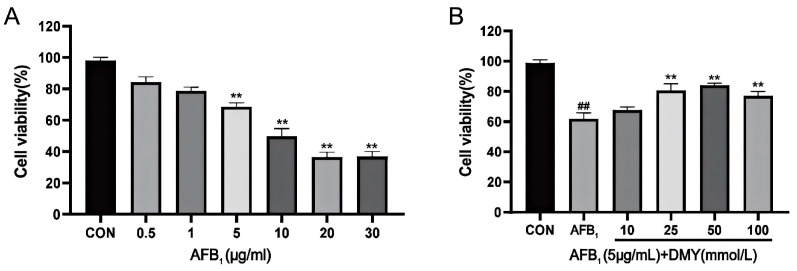
AFB_1_ damaging concentration (**A**) and DMY effective protective concentration (**B**) of MDCK cells. CON, blank control group; AFB_1_, aflatoxin B_1_; DMY, dihydromyricetin. In (**A**), ** indicates *p*  <  0.01 when comparing different AFB_1_ treatment dosage groups with the CON group. In (**B**), ** indicates *p*  <  0.01 when comparing different DMY treatment dosage groups with the AFB_1_ group; ## indicates *p*  <  0.01 when comparing the AFB_1_ group with the CON group.

The CCK-8 method was used to assess the effect of AFB_1_ and DMY on the viability of MDCK cells. As shown in Figure 1A, the viability of MDCK cells exhibited a downward trend with increasing AFB_1_ concentration. When the AFB_1_ concentration exceeded 5 μg/mL, MDCK cell viability was significantly lower than that of the CON group (Figure 1A, *p* < 0.01). Therefore, 5 μg/mL AFB_1_ was selected as the damaging concentration for MDCK cells.

Next, we examined the protective effect of DMY and found that the viability of MDCK cells in the 25, 50, and 100 mmol/L DMY groups was significantly higher than in the AFB_1_ group (Figure 1B, *p* < 0.01). Additionally, the 25 and 50 mmol/L DMY groups exhibited relatively higher cell viability compared to the 100 mmol/L DMY group. Thus, 25 and 50 mmol/L DMY were chosen as the protective concentrations.

### 3.2. DMY Suppressed AFB_1_-Induced Apoptosis and Mitochondrial Membrane Potential Reduction in MDCK Cells

**Figure 2:**
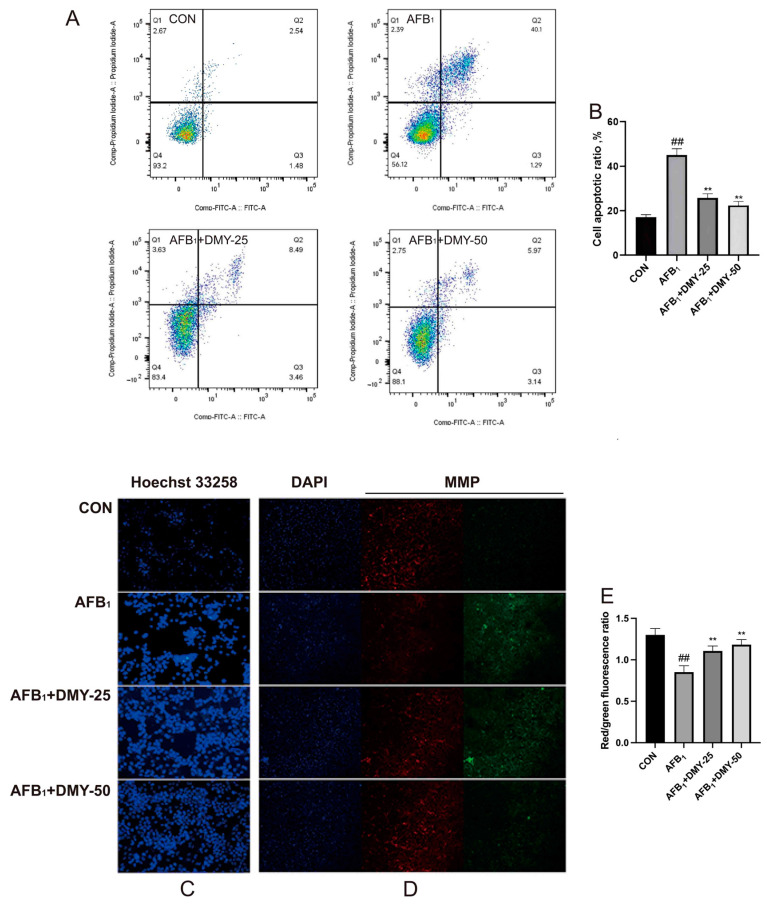
Effect of DMY on AFB_1_-induced apoptosis and reduction in mitochondrial membrane potential in MDCK cells. (**A**,**B**) Cell apoptosis was measured by flow cytometry analysis. (**C**) Hoechst 33258 staining for cell apoptosis. (**D**,**E**) JC-1 staining for mitochondrial membrane potential (MMP) assessment, Scale bar: 20 μm. ** indicates *p*  <  0.01 when comparing different DMY treatment groups with the AFB_1_ group; ## indicates *p*  <  0.01 when comparing the AFB_1_ group with the CON group.

The apoptosis rate of MDCK cells was analyzed by flow cytometry using Annexin V-FITC/PI staining. As illustrated in Figure 2A,B, apoptosis was significantly increased in the AFB_1_ group compared to the CON group (*p* < 0.01). However, DMY treatment effectively attenuated this increase in a dose-dependent manner (*p* < 0.01). These findings were further supported by Hoechst 33258 staining, which revealed marked nuclear condensation and fragmentation in the AFB_1_ group, as indicated by strong fluorescence signals (Figure 2C). In contrast, DMY treatment led to visibly reduced fluorescence intensity, suggesting that DMY exerted a protective effect against AFB_1_-induced apoptosis.

Changes in mitochondrial membrane potential were assessed using JC-1 staining. As shown in Figure 2D, cells treated with AFB_1_ exhibited an increase in green fluorescence and a concomitant decrease in red fluorescence compared to the CON group, indicating a loss of mitochondrial membrane potential. In contrast, treatment with 25 and 50 mmol/L DMY effectively restored mitochondrial function, as evidenced by decreased green fluorescence and increased red fluorescence. Quantitative analysis in Figure 2E further confirmed these observations. AFB_1_ treatment significantly reduced the red to green fluorescence ratio, while treatment with 25 and 50 mmol/L DMY markedly reversed this reduction (*p* < 0.01). These findings indicate that DMY alleviates AFB_1_-induced mitochondrial dysfunction in MDCK cells.

### 3.3. DMY Alleviated AFB_1_-Induced Oxidative Stress in MDCK Cells

**Figure 3:**
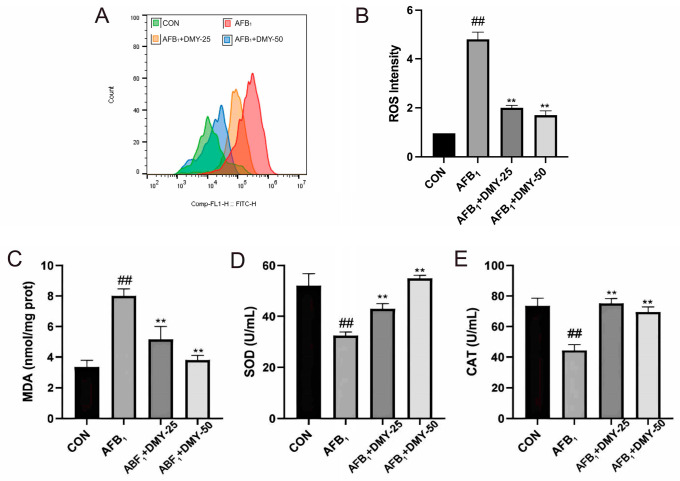
Effect of DMY on AFB_1_-induced oxidative stress in MDCK cells. (**A**,**B**) DCFH-DA fluorescence analysis of reactive oxygen species (ROS) level. (**C**) Malondialdehyde (MDA) content. (**D**) superoxide dismutase (SOD) activities and (**E**) Catalase (CAT). ** indicates *p*  <  0.01 when comparing different DMY treatment groups with the AFB_1_ group; ## indicates *p*  <  0.01 when comparing the AFB_1_ group with the CON group.

The results in Figure 3 showed that AFB_1_ significantly increased ROS and MDA levels while decreasing SOD and CAT activities in MDCK cells compared with the CON group (*p* < 0.01). However, treatment with 25 and 50 mmol/L DMY significantly reduced the ROS and MDA levels and enhanced SOD and CAT activities compared with the AFB_1_ group (*p* < 0.01). These findings suggest that DMY could alleviate AFB_1_-induced oxidative stress in MDCK cells in a dose-dependent manner.

### 3.4. DMY Improved AFB_1_-Induced Inflammation in MDCK Cells

**Figure 4:**
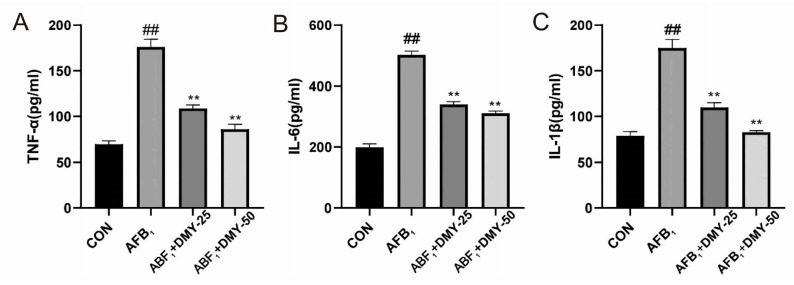
Effect of DMY on AFB_1_-induced inflammation development in MDCK cells. (**A**) Tumor necrosis factor-α (TNF-α), (**B**) interleukin-6 (IL-6), and (**C**) interleukin-1β (IL-1β) levels. ** indicates *p*  <  0.01 when comparing different DMY treatment groups with the AFB_1_ group; ## indicates *p*  <  0.01 when comparing the AFB_1_ group with the CON group.

As shown in Figure 4, AFB_1_ significantly increased the levels of the proinflammatory cytokines TNF-α, IL-6, and IL-1β in MDCK cells compared to the CON group (*p* < 0.01). However, 25 and 50 mmol/L DMY treatment significantly reduced the levels of these cytokines in AFB_1_-treated cells (*p* < 0.01), and the anti-inflammatory effect of DMY became more pronounced with increasing concentration.

### 3.5. DMY Inhibited AFB_1_-Induced Changes in the Expression of Autophagy-Related Proteins in MDCK Cells

**Figure 5:**
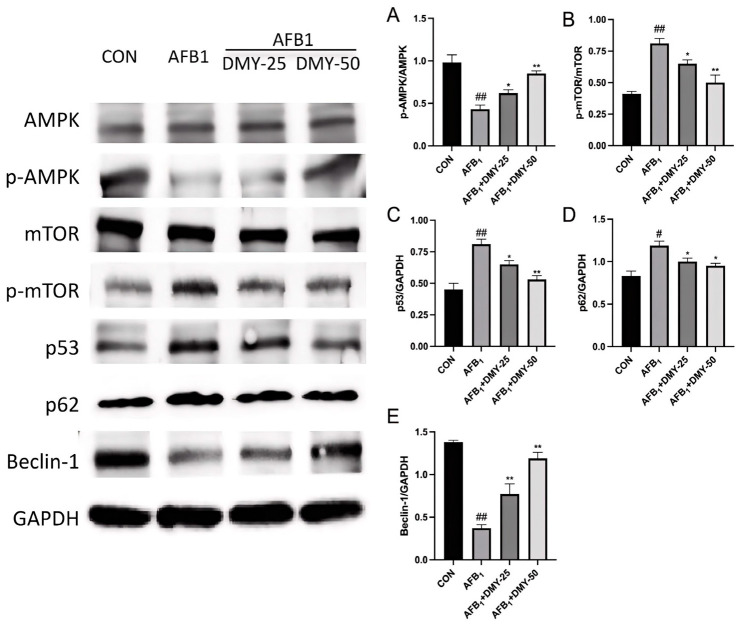
Effect of DMY on AFB_1_-induced expression of autophagy-related proteins in MDCK cells. AMPK, adenosine monophosphate-activated protein kinase; p-AMPK, phosphorylated AMPK; mTOR, mammalian target of rapamycin; p-mTOR, phosphorylated mTOR; GAPDH, glyceraldehyde-3-phosphate dehydrogenase. (**A**) p-AMPK/t-AMPK protein expression ratio; (**B**) p-mTOR/t-mTOR protein expression ratio; (**C**) p53/GAPDH protein expression ratio; (**D**) p27/GAPDH protein expression ratio; (**E**) Beclin-1/GAPDH protein expression ratio. * indicates *p*  <  0.05, and ** indicates *p*  <  0.01 when comparing different DMY treatment groups with the AFB_1_ group; # *p*  <  0.05, and ## indicates *p*  <  0.01 when comparing the AFB_1_ group with the CON group.

As illustrated in Figure 5, AFB_1_ inhibited the expression of the autophagy-related proteins Beclin-1, while promoting the expression of p53 and p62 in MDCK cells compared to the CON group (*p* < 0.05). In contrast, treatment with 25 and 50 mmol/L DMY effectively upregulated the expression of Beclin-1, while downregulating the expression of p53 and p62 relative to the AFB_1_ group (*p* < 0.05). Additionally, the p-AMPK/AMPK ratio in the AFB_1_ group was significantly lower than that in the CON group (*p* < 0.01), whereas the p-mTOR/mTOR ratio was significantly higher (*p* < 0.01), indicating suppressed autophagic activity. Following treatment with 25 and 50 mmol/L of DMY, the p-AMPK/AMPK ratio significantly increased (*p* < 0.05), while the p-mTOR/mTOR ratio significantly decreased (*p* < 0.05), suggesting that DMY restored AFB1-inhibited autophagy in MDCK cells.

## 4. Discussion

Animal feeds and their raw materials are susceptible to *A. flavus* contamination during outdoor drying and storage, leading to substantial aflatoxin accumulation over time. As early as 1993, AFB_1_ was categorized as a class I carcinogen by the IARC [22]. To date, more than 20 aflatoxins and their derivatives have been identified, with AFB_1_ being the most abundant and widely distributed [23]. The toxic effects of AFB_1_ are well documented, with chronic exposure in farm animals causing liver and kidney damage, immunosuppression, and increased oxidative stress [24]. Consequently, there is growing interest in natural pharmaceutical compounds capable of alleviating or preventing AFB_1_-induced toxicity, in line with current industry trends.

The MDCK cells used herein possess typical renal epithelial properties (e.g., cytokeratin expression) and stable, mycoplasma-free growth; their selection was justified by avoiding interspecies differences (reflecting canine renal responses), wide validation in renal toxicology, and sensitivity to nephrotoxins—making them ideal for evaluating DMY’s protective effects. The 5 μg/mL AFB1 concentration used in this study, comparable to the medium-high AFB1 contamination level in commercial dog foods [11], induces significant cellular damage without causing complete cell death—consistent with the pathological process of chronic kidney injury from long-term low-dose AFB1 exposure in dogs—and when co-treated with 10–100 mmol/L DMY (most effective at 25 and 50 mmol/L) improved MDCK cell viability (which declined notably at AFB1 concentrations >5 μg/mL), leading to the selection of 5 μg/mL AFB1 and 25/50 mmol/L DMY as optimal damaging and protective concentrations, respectively, and enhancing the clinical relevance of DMY’s protective effect.

Organisms possess a comprehensive antioxidant system to maintain the balance of free radical metabolism, and disruptions in this balance are indicative of oxidative damage in cells [25]. The degree of oxidative damage can be assessed by detecting apoptosis and the accumulation of ROS [26]. ROS serve as critical upstream signals that trigger the onset of apoptosis and effectively stimulate the expression of the autophagy receptor protein p62, thereby promoting further apoptotic signaling [27,28]. AFB_1_ can exacerbate oxidative stress and impair the oxidative phosphorylation process by inducing ROS overproduction in renal cells, leading to reduced cell viability, activation of the apoptotic pathway, and ultimately, a self-perpetuating cycle of cell damage [29]. In this study, SOD and CAT activities, along with ROS and MDA levels, were used to assess oxidative injury in MDCK cells, while TNF-α, IL-6, and IL-1β levels were measured to evaluate inflammation. The results showed that DMY significantly reversed the AFB_1_-induced reductions in SOD and CAT activities, the increases in MDA and ROS levels, and the elevated levels of inflammatory cytokines TNF-α, IL-6, and IL-1β, thereby inhibiting apoptosis in MDCK cells. These findings are consistent with those of Wu et al., who reported that DMY inhibits apoptosis by enhancing SOD activity and reducing MDA levels and inflammatory cytokines (IL-1, IL-6, TNF-α, and MCP-1) in mouse kidneys [19]. This suggests that DMY can attenuate cellular oxidative stress by modulating oxidative metabolic pathways, effectively reducing AFB_1_-induced apoptosis in MDCK cells.

ATP homeostasis in mammalian cells is closely associated with cell growth, proliferation, autophagy and apoptosis [12]. AMPK, a key regulator of ATP balance, is widely distributed in metabolic organs and plays an important role in maintaining cellular energy status, metabolism function, and apoptosis regulation in vivo [30,31]. Autophagy is a tightly regulated process. The tumor suppressor gene p53 promotes autophagy by upregulating upstream regulators of mTOR through a transcription-dependent mechanism [32]. mTOR, a serine/threonine protein kinase, negatively regulates autophagy, while p62 functions as a selective autophagy substrate that accumulates when autophagy is impaired [33,34]. Therefore, p53 and p62 levels are commonly used as indicators of autophagy inhibition. Beclin-1, another key autophagy-related protein, regulates both the initiation and maturation of autophagosomes [35]. In this study, AFB_1_ was found to decrease the p-AMPK/AMPK ratio, increase the p-mTOR/mTOR ratio, promote p53 expression, and suppress Beclin-1 expression in MDCK cells. These results are consistent with those of Chen et al., who reported that AFB_1_ induces apoptosis in Leydig cells by reducing the p-AMPK/AMPK ratio and increasing the p-mTOR/mTOR ratio [36]. They also found that AFB_1_ upregulated the autophagy related protein p62, while downregulating the anti-apoptotic protein Bcl-2 [36]. Similar findings were reported in another study, which demonstrated that AFB_1_ decreased Beclin-1 and increased p53 mRNA expression levels [37]. These effects may be linked to AFB_1_-induced lysosomal dysfunction [38]. Specifically, AFB_1_ promotes lysosomal alkalinization, leading to lysosomal membrane permeabilization [39]. As a result, autophagosome degradation is impaired, causing a blockade at the late stage of autophagic flux [39,40]. This ultimately leads to p62 accumulation and suppression of Beclin-1 expression. However, when DMY was added, autophagy-related markers were significantly improved. This suggests that modulation of autophagy signaling is one of the mechanisms by which DMY alleviates AFB_1_-induced apoptosis in MDCK cells.

The mitochondrial pathway is a classical mechanism of apoptosis, with the anti-apoptotic protein Bcl-2 playing key roles in maintaining mitochondrial structural integrity and functional stability [41]. The mitochondrial membrane potential is essential for ATP production and for sustaining the tricarboxylic acid cycle and oxidative phosphorylation; a decrease in this potential is indicative of cellular damage [42]. Our study showed that DMY ameliorated the AFB_1_-induced increase in p53, p62, and p-mTOR expression in MDCK cells, thereby effectively enhancing mitochondrial membrane potential. This suggests that DMY mitigates AFB_1_-induced cellular injury and contributes to the maintenance of renal cell homeostasis. These findings are similar to those of Guo et al., who reported that DMY exerts protective effect against fatty liver in a rat model by suppressing the protein expression of NF-κB, p53 [43,44]. It indicates that DMY can effectively alleviate AFB_1_-induced oxidative stress and apoptosis in MDCK cells at the cellular level. However, further research is required to elucidate its protective effects at the organismal level in canines.

A key translational consideration for our in vitro findings-regarding DMY’s protection against AFB1-induced MDCK cell injury—lies in DMY’s pharmacokinetic properties. DMY exhibits poor oral bioavailability (<10% in rodent models) due to high hydrophilicity limiting intestinal permeability, and rapid metabolism: it undergoes extensive phase II glucuronidation in the intestines/liver, forming inactive metabolites that are quickly excreted, resulting in a short in vivo half-life (1 to 2 h in mammals) [16,17,19]. These traits mean free oral DMY may fail to maintain therapeutic concentrations in canine renal tissues, potentially weakening its efficacy against chronic AFB1-induced kidney injury. Our study focuses on elucidating DMY’s cellular protective mechanism (using MDCK cells to reflect canine renal responses), but addressing these pharmacokinetic limitations is critical for future in vivo translation. As supported by DMY’s established renal protective potential in preclinical models [19,20], subsequent canine studies should prioritize optimizing DMY delivery: nanoparticle/liposomal encapsulation to enhance stability and permeability, or co-administration with absorption enhancers (e.g., piperine) to inhibit glucuronidation. Additionally, canine-specific pharmacokinetic studies are needed to define dosing regimens (frequency, dosage) that sustain DMY levels in kidneys, accounting for species differences in hepatic metabolism.

## 5. Conclusions

At the cellular level, DMY ameliorates AFB1-induced injury in MDCK cells by enhancing anti-inflammatory and antioxidant capacities, restoring mitochondrial oxidative phosphorylation, and modulating the autophagy pathway. These findings provide a theoretical basis for the potential therapeutic application of DMY in treating canine renal damage induced by AFB1-contaminated diets. Beyond canine health, the results highlight DMY as a promising natural compound for mitigating mycotoxin-induced toxicity in other livestock and companion animals, given the widespread occurrence of AFB1 contamination in global animal feeds. Furthermore, DMY’s multifaceted protective mechanisms support its potential development as a functional additive in pet food or animal feed to improve food safety and reduce mycotoxin-related health risks. Future studies should validate these effects in in vivo canine models to determine optimal dosages and delivery methods for practical application.

## Data Availability

The data presented in this study are available on request from the corresponding author. The data are not publicly available due to privacy.
